# Time to be “smart”—Opportunities Arising From Smartphone-Based Behavioral Analysis in Daily Patient Care

**DOI:** 10.3389/fnbeh.2018.00303

**Published:** 2018-12-04

**Authors:** Kevin Akeret, Flavio Vasella, Olivia Geisseler, Noemi Dannecker, Arko Ghosh, Peter Brugger, Luca Regli, Martin N. Stienen

**Affiliations:** ^1^Department of Neurosurgery, University Hospital Zurich & Clinical Neuroscience Center, University of Zurich, Zurich, Switzerland; ^2^Laboratory of Molecular Neuro-Oncology, Clinical Neuroscience Center, Department of Neurology, University Hospital Zurich, Zurich, Switzerland; ^3^Neuropsychology Unit, Department of Neurology, University Hospital Zurich, University of Zurich, Zurich, Switzerland; ^4^Cognitive Psychology Unit, Institute of Psychology, Leiden University, Leiden, Netherlands

**Keywords:** smart surgery, smartphone, neuropsychology, behavioral analysis, digital behavior, smartphone-based monitoring, machine learning

## Abstract

While pathologies of the central nervous system (CNS) are often associated with neuropsychological deficits, adequate quantification and monitoring of such deficits remains challenging. Due to their complex nature, comprehensive neuropsychological evaluations are needed, which are time-consuming, resource-intensive and do not adequately account for daily or hourly fluctuations of a patient’s condition. Innovative approaches are required to improve the diagnostics and continuous monitoring of brain function, ideally in the form of a simple, objective, time-saving and inexpensive tool that overcomes the aforementioned weaknesses of conventional assessments. As smartphones are widely used and integrated in virtually every aspect of our lives, their potential regarding the acquisition of data representing an individual’s behavior and health is enormous. Alterations in a patient’s physical or mental health state may be recognized as behavioral deviation from the physiological range of the normal population, but also in comparison to the patient’s individual baseline assessment. As smartphone-based assessment allows for continuous monitoring and therefore accounts for possible fluctuations or transiently occurring abnormalities in a patient’s neurologic state, it may serve as a surveillance tool in the acute setting for early recognition of complications, or in the long-term outpatient setting to quantify rehabilitation or disease progress. This may be particularly interesting for regions of the world where healthcare resources for comprehensive clinical/neuropsychological examinations are insufficient or distances to healthcare providers are long. Here, we highlight the potential of smartphone-based behavioral monitoring in healthcare.

**Clinical Trial Registration**: www.clinicaltrials.gov, identifier NCT03516162.

Pathologies of the central nervous system (CNS) are commonly associated with neurological and/or neuropsychological deficits, either due to the disease itself or as inadvertent sequelae of neurosurgical treatment, in case this is required. Both their diagnosis and surveillance are crucial to initiate rehabilitative treatment and to evaluate recovery over time. While focal neurological deficits can be detected relatively easy, most neurosurgeons—as well as other physicians in clinical neuroscience—have more difficulties in detecting, accurately quantifying and monitoring neuropsychological dysfunction over time. Reasons for this include the complex nature of neuropsychological deficits, typically encompassing multiple domains such as attention, memory, executive functions or language, as well as their fluctuating character. This represents a significant shortcoming, as the prevalence of neuropsychological salience in many CNS pathologies outweighs that of focal neurological deficits by far (Tucha et al., 2000; Goebel and Mehdorn, 2011; Stienen et al., 2013). Comprehensive neuropsychological assessments have therefore become integral parts of the pre- and postoperative protocols in many specialized neurosurgical departments (Zweifel-Zehnder et al., 2015).

While the standardized use of neuropsychological assessments has positively impacted on patient care, a number of significant limitations remain. First, routinely used screening instruments such as the Montréal Cognitive Assessment (MoCA) are simple tools with ceiling effects that do not sufficiently account for subtle changes in function, particularly in patients that perform relatively well. Second, comprehensive neuropsychological evaluations are quite time-consuming (with interviews taking up to 3 h) and resource-intensive, as they require specialized personnel and patient transfer to the hospital for the face-to-face assessment. In illiterates or foreign-language patients, those assessments are impossible or invalid. Third, assessments only represent the momentary functional status, typically evaluated once before and several weeks after surgical treatment, not accounting for daily or hourly fluctuations of a patient’s condition.

Innovative approaches would be desired to improve the diagnostics and continuous monitoring of brain function, ideally in the form of a simple, objective, time-saving and inexpensive tool that overcomes the aforementioned weaknesses of conventional assessments without sacrificing accuracy and negatively impacting quality of patient care.

The vast majority of adults in developed countries owns a smartphone nowadays (Althoff et al., 2017). Whether at work, during spare time or for social interactions, smartphones are integrated in virtually every aspect of our lives, having become a mirror of our behavior. Their potential regarding health issues is only beginning to be recognized, but is already reflected in the large number of available health-related applications for individual amateur self-assessment (Servick, 2015). In addition, smartphones are becoming more and more implemented in professional medical assessments, e.g., for cardio-respiratory monitoring (Sohn et al., 2017) psychiatric assessment (Egger et al., 2018) or modification of dietary habits (Recio-Rodriguez et al., 2018).

The widespread and frequent use of smartphones enables the acquisition of vast amounts of data representing an individual’s behavior. Physical activity level has for example been analyzed on a worldwide basis using metadata from smartphones (Althoff et al., 2017) and this may only be the beginning: frequency and timing of smartphone use, as well as the speed of touchscreen tapping deliver information that reflect an individual’s functional level in real-time (Figure 1). Alterations in a patient’s physical or mental health state may be recognized in two different ways: (1) as behavioral deviation from the physiological range of the normal population, but also (2) in comparison to the patient’s individual baseline assessment before disease onset/surgery. The smartphone-based assessment allows for continuous monitoring, therefore accounting for both robust changes in a patient’s smartphone-based behavior, but also for abnormalities that only occur transiently.

**Figure 1:**
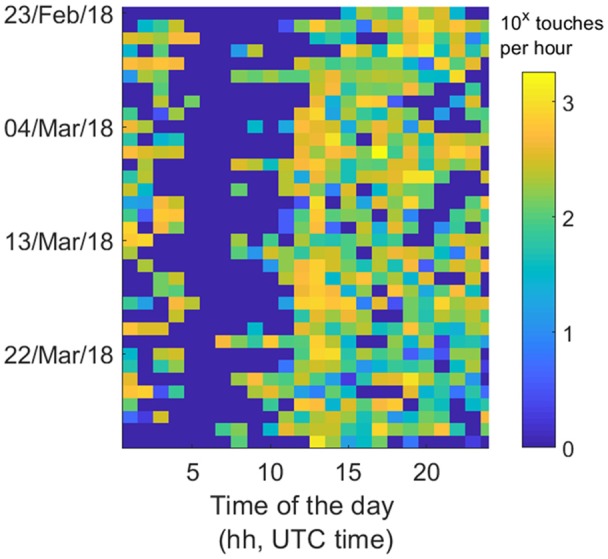
Example of data collected using a smartphone application that tracks the touchscreen interactions. Smartphones can occupy the user throughout the day. These parameters can extend from how quickly people tap on the phone to fitting circadian models. In this example, the heatmap depicts touchscreen taps per hour over the course of a month, where each row represents a specific date and each column represents the time of the day. By monitoring the touchscreen interactions in the background, a range of parameters can be derived to quantify behavioral alterations. These data were collected from a patient located in the Netherlands; the data are displayed in UTC time.

Applications allowing the collection of such data have been developed and are available on the market. There are on-going clinical studies that critically analyze the value of smartphone-based behavioral monitoring in neurosurgical patients pre-, peri- and postoperatively. Our team, for example, is currently enrolling patients into the prospective, observational “*Smart Surgery Study*,” registered under http://www.clinicaltrials.gov (Identifier: NCT03516162). The aim of this study is to determine how a patient’s digital behavior or smartphone usage correlates with neurological and neuropsychological function before and after routine neurosurgical operations. Studies such as the “*Smart Surgery Study*” will allow us to establish the value of smartphone-based behavioral monitoring, or lack thereof, in the near future.

Many advantages could conceivably arise from the introduction of such a tool in daily routine. Smartphone-based evaluations are convenient for both the patient (as they may reduce the number of clinical examinations) and the care-provider (as they may reduce personnel and hospital expenses). Apart from its application in clinical practice, smartphone-based assessments open a venue to overcome current weaknesses in healthcare research: the active and usually subjective determination of patient outcome at predefined time points, inherent to inter- and intra-observer variability, false grading on outcome scales, or missing data. Here, smartphones offer completely objective, passive and unobtrusive acquisition of longitudinal data (Figure 1). Thanks to impressive advances in artificial intelligence and machine learning, large amounts of behavioral “big data” can be handled and sophistically interpreted today. Despite all these potential benefits, it is of course crucial that ethical issues regarding data acquisition in such a setting are addressed from the beginning and that the risk of data abuse is minimized (Kreitmair et al., 2017). Both patients and health professionals need to be confident that the highly sensitive personal data is safe for smartphone-based monitoring to be implemented routinely in healthcare (Proudfoot et al., 2010; Dehling et al., 2015). Tackling technical issues such as the reduction of smartphone battery-life is equally important to not disincentivize the use of applications allowing for health-related monitoring (Dennison et al., 2013). Evidently, however refined and intricate these technologies may become, the value of direct patient interaction cannot be overstated, and it is highly unlikely that an evaluation by an experienced health professional will be replaced by digital behavior analysis in the foreseeable future. Both aspects of health-care should act closely intertwined—the smartphone-based monitoring as means to screen patients for changes in behavior, which can subsequently be addressed by a dedicated workup from experienced healthcare professionals.

In conclusion, smartphone-based behavioral monitoring has significant potential in healthcare, in particular in the field of neuroscience. The evaluation of brain function may become more sensitive and adequate by accounting for its intrinsic complexity and fluctuations. In the future, smartphone-based monitoring may serve as a surveillance tool in the acute setting for early recognition of complications, or in the long-term outpatient setting to quantify rehabilitation or disease progress. It may especially hold promise for rural or developing regions where healthcare resources for neuropsychological examinations are scarce or distances to healthcare providers are long. While the implementation of this type of behavioral monitoring gives rise to several challenges that need to be addressed proactively, leveraging the possibilities arising from widespread smartphone use may ultimately improve patient care, if we are “smart” about it.

## Ethics Statement

The “Smart Surgery Study” is carried out in accordance with the recommendations of the Kantonale Ethikkommission Zürich (KEK-ZH). The protocol was approved by the KEK-ZH under the case number BASEC 2018–00395. All subjects give written informed consent in accordance with the Declaration of Helsinki.

## Author Contributions

MS, LR, AG, PB and OG contributed to the conception and design of the study. AG and MS organized the databases. MS organized IRB-approval and trial registration. KA, FV, OG, ND and MS recruit patients and acquire data. MS, KA, FV, OG, ND, PB and AG perform the statistical analysis. KA and FV wrote the first draft of the manuscript. MS and AG wrote sections of the manuscript. All authors contributed to manuscript revision, read and approved the submitted version.

## Conflict of Interest Statement

AG is an inventor of the technology used to track touchscreen interactions in this study. AG is co-founder of QuantActions GmbH, a company focused on quantifying human behavior through smartphone interactions. The remaining authors declare that the research was conducted in the absence of any commercial or financial relationships that could be construed as a potential conflict of interest.
